# Effect of *Rhododendron arboreum *Leaf Extract on the Antioxidant Defense System against Chromium (VI) Stress in *Vigna radiata *Plants

**DOI:** 10.3390/plants9020164

**Published:** 2020-01-29

**Authors:** Vandana Gautam, Pooja Sharma, Palak Bakshi, Saroj Arora, Renu Bhardwaj, Bilal Ahmad Paray, Mohammed Nasser Alyemeni, Parvaiz Ahmad

**Affiliations:** 1 Department of Botanical and Environmental Sciences, Guru Nanak Dev University, Amritsar 143005, India; vndu.gndu@gmail.com (V.G.); pooja.sharma@gmail.com (P.S.); p.bakshi@gmail.com (P.B.); sarojarora.gndu@gmail.com (S.A.); renubhardwaj82@gmail.com (R.B.); 2 Zoology Department, College of Science, King Saudi University, Riyadh 11451, Saudi Arabia; bparay@ksu.edu.sa; 3 Botany and Microbiology Department, College of Science, King Saudi University, Riyadh 11451, Saudi Arabia; mnyemeni@ksu.edu.sa; 4 Department of Botany, S.P. College, Srinagar 190001, India

**Keywords:** *Rhododendron arboreum*, *Vigna radiata*, enzymes activity, chromium (Cr), polyphenols

## Abstract

In the current investigation, we studied role of *Rhododendron* leaf extract in *Vigna radiata* grown under chromium metal stress. We observed that seed treatment with *Rhododendron* leaf extract resulted in the recuperation of seedling growth under chromium toxicity. Seed treatment with *Rhododendron* leaf extract significantly improved the contents of anthocyanin and xanthophyll pigments under stress. The antioxidative defense system triggered after *Rhododendron* extract treatment, resulting in the increased actions of antioxidant enzymes. Oxidative stress induced by the assembly of reactive oxygen species was reduced after *Rhododendron* extract treatment under chromium toxicity as indicated by the enhanced contents of non-enzymatic antioxidants, namely ascorbic acid, tocopherol, and glutathione. Furthermore, *Rhododendron* leaf extract treatment under chromium metal stress also encouraged the biosynthesis of organic acids, polyphenols, as well as amino acids in *Vigna radiata*. Statistical analysis of the data with multiple linear regression also supported that *Rhododendron* leaf extract can effectively ease chromium metal-induced phytotoxicity in *Vigna radiata*.

## 1. Introduction

Heavy metals, put forth by the modern urbanized and industrialized environment, are major chemical agents causing hindrances in normal growth, functioning, productivity, and survival of plants as well as animals [1]. In recent times, heavy metal toxicity in farming systems along with edible crops has become a major concernnot only from an environmental but also from a health point of view. Chromium (Cr) is the prevalent element, whichexists in different oxidation states in nature, amongst which, the hexavalent chromium [Cr(VI)] is the most pernicious [2]. Volcanic dust and igneous rocks are the natural sources of chromium; however, agricultural land is severely contaminated with chromium metal by inappropriate waste dumping or seepage from tanneries and burning of coal or oil [3,4]. Usually, chromium is not persistent in the atmosphere as it is deftly deposited in soil or dissolved in water. Therefore, it is readily available to the roots of plants budding in the infected region. Even in low concentrations, Cr is reported to enhance the production of reactive oxygen species (ROS), including hydrogen peroxide (H_2_O_2_) and superoxide anion,which lead to oxidative stress [5]. Oxidative stress hinders seed germination, growth, and metabolism in plants along with cell injury. Living organisms are equipped with an antioxidant defense system against ROS, which consists of enzymatic and non-enzymatic antioxidants [6]. Antioxidant enzymes include superoxide dismutase (SOD), catalase (CAT), ascorbate peroxidase (APOX), glutathione peroxidase (GPOX), guaiacol peroxidase (POD), polyphenol oxidase (PPO), dehydroascorbate reductase (DHAR), glutathione-S-transferase (GST), and glutathione reductase (GR) [7]. Non-enzymatic antioxidants comprise of ascorbic acid, tocopherol, glutathione (GSH), and polyphenols. Tocopherol repairs peroxyl radicals and terminates the chain of lipid peroxidation [8,9]. Thus, the antioxidative defense system helps to overcome oxidative stress. However, this system does not work efficiently when living organisms encounter severe metal stress, resulting in a need for external supplements to beat the oxidative stress.

A literature survey reveals the oxidative stress protective and plant growth regulatory effects of several plant extracts. For example, tea seed extract has been reported to regulate the growth of *Brassica *sp., *Avena sativa*, and *Hordeum vulgare *[10]. *Rhododendron arboreum* of the Ericaceae family is native to Bhutan, China, India, and Nepal. The trees grow in North and North-East India. The name ‘rhododendron’ is derived from the Greek word ‘rhodo’ meaning rose and ‘dendron’ meaning tree. *Rhododendron *is the national flower of Nepal and the state tree of Uttarakhand (India). This evergreen tree is important from an economical as well as a horticultural perspective. Also, it is widely used by the tribal people of North India for gastronomic as well as traditional restorative purposes. In our previous study [11], its antioxidant and antimutagenic properties were evaluated along with the active principals responsible for those activities. It has also been reported to be a resource of a number of phytoconstituents of therapeutic value by numerous authors [12,13,14]. So, keeping in view the eco-friendly and remunerative nature of plant extracts, the present study was designed to evaluate the Cr metal stress-ameliorating activity of *Rhododendron arboreum* leaves using *Vigna radiata*.

Due to its high protein content and low price, *Vigna radiata* has become the most widespread food item in every household all over the globe. In India, it is frequently used as food, especially for babies, old, and sick people, because of its easy digestibleness and great nutritional value. Its fiber is also an outstanding feed for farm animals. In addition, the capability of *Vigna radiata* to fix atmospheric nitrogen and add organic matter to the soil are major characteristics to maintain soil fertility. It has been previously reported that Cr exposure has harmful effects on the physiological and biochemical processes of *Vigna radiata* L [15]. Furthermore, metal stresses decrease the productivity of crops and go through the food chain, resulting in terrible health problems to the consumers, including human beings and other herbivores [16]. The *Vigna radiata *crop was selected for the present research since it fits well as a model because of its short life cycle and low maintenance nature.

The current analysis was carried out to investigate the effect of methanol extract of *Rhododendron arboreum* leaves (MEL) on Cr-treated *Vigna radiata* plants. The analyzed biochemical parameters included anthocyanin and xanthophyll pigment contents, antioxidant enzymes estimation, non-enzymatic antioxidants (ascorbic acid, tocopherol, and glutathione) analysis, and organic acids, polyphenols, and amino acids analysis.

## 2. Materials and Methods

The stress protective effect of methanol extract of *Rhododendron arboreum* leaves (MEL) on Cr(VI)-treated *Vigna radiata* plants was observed by analyzing the pigment contents, protein content, enzymatic antioxidants, non-enzymatic antioxidants, polyphenols, amino acids, and organic acids.

### 2.1. Raising of Plant Material

The certified seeds of *Vigna radiata* var. SML-668 were procured from Punjab Agricultural University, Ludhiana (India). The healthy seeds of *Vigna radiata* were surface sterilized for one minute with 0.01% mercuric chloride (HgCl_2_) and rinsed with distilled water several times. The seeds were given a pre-sowing treatment with different concentrations (125, 250, and 500 ppm) of *Rhododendron* leaf extract for six hours. Petriplates lined with whatman filter paper 1 were supplemented with 250 μM chromium (IV). These solutions were prepared in distilled water. The plants were grown in petriplates kept in a seed germinator for 7 days under controlled conditions of 25±0.5 °C temperature, 16-h photoperiod, 8-h dark period, 175 μmol/m^2^/s light intensity, and 60% relative humidity. After 7 days of growth, the plants were supplemented with half-strength Hoagland medium and the petriplates were monitored for 7 days, after which the plants were harvested and independent experiments were performed. Each petriplate contained 20 seeds and all the treatments were given in triplicates in order to ensure valid statistical analysis.

### 2.2. Rhododendron arboreum Methanol Leaf Extract(MEL)

The fresh leaves of* Rhododendron arboreum* leaves were washed with double-distilled water, dried in the shade, and ground to a fine powder in a mixergrinder. Them, 1 kg of powder was extracted with 80% methanol and dried in a vacuum rotary evaporator to get 68.38 g (6.83%) of methanol leaf extract. The nontoxic and maximum yield producing (in terms of the root length, shoot length, fresh weight, and dry weight) concentrations of the MEL (methanol extract of *Rhododendron arboreum* leaves) were identified by carrying out different test experiments on *Vigna radiata* seeds. Three concentrations, i.e., 125, 250, and 500 ppm, were selected for the present study. A 1000 ppm mother stock of MEL was prepared and working concentrations of 125, 250, and 500 ppm were prepared from the mother stock by serial dilutions. The mother stock and working concentrations were stored in a refrigerator at 4 °C.

### 2.3. Cr(VI)Metal

Heavy metal Cr(VI) was given in the form of potassium chromate (K_2_CrO_4_) (Qualigens Fine Chemicals Pvt. Ltd., Mumbai, India). The IC_50_ value (250 μM) of the metal was determined and used for the treatments. The working 250 μM concentration of Cr(VI) was prepared from 10 mM mother stock.

### 2.4. Treatments

All the treatments given in the present study are shown in Table 1.

### 2.5. Phenolic Pigments:

#### 2.5.1. Xanthophyll Content

The plant tissue was tested for xanthophyll content using the method of Lawrence [17]. Hexane, acetone, absolute alcohol, and toluene were mixed in the ratio of 10:7:6:7 to prepare the extractant. Then, 50 mg of dried plant sample was converted into a fine powder using a pestle and mortar. The powdered plant sample was mixed with 30 mL of extractant solution in a volumetric flask and shaken for 10–15 min. The hot saponification process was started by adding 2 mL of methanolic KOH (40%). The contents were shaken for 1–2 min, then the flask was kept at 56 °C in a water bath followed by incubation for one hour in cool dark conditions. Then, 30 mL of hexane was added to the reaction mixture with continued vigorous shaking and the final volume of 100 mL was made with the addition of 10% Na_2_SO_3_. The flask was again incubated for one hour in dark conditions. The absorbance (A) of the upper phase was noted at 474 nm.

#### 2.5.2. Anthocyanin Content

The anthocyanin content of the fresh plant sample was estimated by the method given by Mancinelli [18]. Under chilled conditions, 1g of fresh plant sample was crushed using a pestle and mortar, in an extraction mixture, which consisted of methanol, distilled water, and hydrochloric acid in a ratio of 79:20:1. The crushed tissue was centrifuged for 20 min at 13,000 rpm and 4 °C. The A of the supernatant was recorded at 530 and 657 nm.

### 2.6. Protein Content

The protein content of the samples was determined by following the method given by Lowry et al. [19]. Onegram of fresh plant samples was weighed and crushed in 3 mL of 50 mM potassium phosphate buffer with pH 7.0 using a pestle and mortar under ice cold conditions. The homogenates were centrifuged for 20 min at 13,000 rpm and 4 °C. In total, 100 µL of supernatant was mixed with 900 µL of distilled water followed by the addition of 5 mL of reagent C. The test tube with 1 mL of distilled water served as a blank. This mixture was mixed well and allowed to stand for 10 min followed by the addition of 500 µL of Folin–Ciocalteau reagent. The reaction mixture was mixed thoroughly and incubated at room temperature for 30 min in dark conditions. The optical density of the blue color was measured at 660 nm. A standard curve of the protein solution representing concentration vs. absorbance was plotted and a linear regression equation was obtained, which was used to calculate the protein content of the samples, which was expressed as mg g^−1^fresh weight.

### 2.7. Enzymatic Antioxidants

#### 2.7.1. Preparation of Plant Extracts

Onegram of fresh plant sample was weighed and crushed in 3 mL of extractant buffer using a pestle and mortar under ice cold conditions. The homogenates were centrifuged for 20 min at 13,000 rpm and 4°C. The supernatant was collected for analysis of the biochemical parameters. The extractant buffer for the estimation of the activities of (POD, CAT, GR, APOX, DHAR, and GPOX) enzymes was 50 mM potassium phosphate buffer with pH 7.0. For estimation of the SOD activity, samples were homogenized in 50 mM sodium carbonate buffer at pH 10.2. The homogenates for GST were prepared in 0.2 M potassium phosphate buffer with pH 7.4 whereas for PPO, 0.1 M potassium phosphate buffer at pH 7.0 was used.

#### 2.7.2. Guaiacol Peroxidase (POD, EC. 1.11.1.7)

The guaiacol peroxidase activity was estimated by method given by Pütter [20]. To prepare 3 mL of the reaction mixture 50 mM phospahte buffer, 20.1 mM of guaiacol solution, 12.3 mM of H_2_O_2_, and 60μL of enzyme extract were added to a cuvette. The rate of the oxidation of guaiacol and formation of guaiacol dehydrogenation product (GDHP) was recorded at 436 nm for 1 min at 3-s intervals at 25 °C. One unit of enzyme activity is defined as the amount of enzyme necessary to catalyze the oxidation of guaiacol and the development of 1 μM of GDHP min^−1^g^−1^fresh weight. The enzyme activity was expressed as UA mg g^−1^ protein

#### 2.7.3. Catalase (CAT, EC 1.11.1.6)

The estimation of the catalase activity was performed according to the method of Aebi [21]. First,3 mL of the reaction mixture consisted of 50 mM phosphate buffer, 15 mM H_2_O_2_, and 60 μL of enzyme extract. The breakdown of hydrogen peroxide caused a decline in the optical density, which was recorded for one minute at 240 nm and 25 °C. The quantity of enzyme necessary to release half peroxide oxygen from hydrogen peroxide is referred to as one unit of the enzyme activity and was expressed as UA mg g^−1^ protein

#### 2.7.4. Superoxide Dismutase (SOD, EC 1.15.1.1)

The superoxide dismutase activity of the fresh plant samples was estimated by the method proposed by Kono [22]. To estimate the SOD activity, a reaction mixture containing 50 mM sodium carbonate buffer, 24 µM Nitro Blue Tetrazolium (NBT), 0.1 mM ethylenediaminetetraacetic acid (EDTA), and 0.03% Triton X-100 was put in a cuvette and the reaction was started by the addition of 1 mM hydroxylamine hydrochloride. Then, 70 µL of enzyme extract were added to the reaction mixture and the increase in the absorbance was observed at 560 nm for 2 min at 6-s intervals at 25 °C. The amount of enzyme required to inhibit NBT reduction up to 50% is defined as one unit of enzyme and was expressed as UA mg g^−1^ protein

#### 2.7.5. Ascorbate Peroxidase (APOX, EC. 1.11.1.11)

The APOX activity of plant samples was estimated by following the method given by Nakano and Asada [23]. First, 50 mM phosphate buffer, 0.5 mM ascorbate, 1 mM H_2_O_2_, and 70 μLof enzyme extract comprised the 3 mL reaction mixture. The decrease in absorbance was recorded at 290 nm. One unit of ascorbate peroxidase was estimated by measuring the amount of enzyme required to oxidize 1 μM of ascorbate min^−1^ g^−1^fresh weight. The APOX activity was expressed as UA mg g^−1^ protein

#### 2.7.6. Glutathione Reductase (GR, EC 1.6.4.2)

The glutathione reductase activity of fresh plant samples was assayed by using the method proposed by Carlberg and Mannervik [24]. The 3-mL reaction mixture in the cuvette consisted of 50 mM phosphate buffer, 1 mM EDTA, 0.1 mM reduced nicotinamide adenine dinucleotide phosphate (NADPH), 1 mM glutathione disulphide, and 75 μL of enzyme extract. The decrease in absorbance per minute was observed at 340 nm at intervals of 3 s at 25 °C. One unit of enzyme activity is defined as the amount of enzyme required to oxidize 1.0 μM of NADPH min^−1^ g^−1^ fresh weight. The activity was expressed as UA mg g^−1^ protein.

#### 2.7.7. Dehydroascorbate Reductase (DHAR, EC. 1.8.5.1)

The dehydroascorbate reductase activity of plant samples was estimated using the method given by Dalton et al. [25]. The 3-mL reaction mixture contained 50 mM phosphate buffer, 0.1 mM EDTA, 1.5 mM reduced glutathione and 0.2 mM dehydroascorbate, and 75 μL of enzyme extract. An increase in the absorbance per minute was noted at 265 nm at 3-s intervals and 25 °C. One unit of dehydroascorbate reductase is defined as the quantity of enzyme needed to catalyze the development of 1 μM of ascorbate min^−1^ g^−1^ fresh weight of plant tissue. The DHAR activity was indicated by UA mg g^−1^ protein

#### 2.7.8. Polyphenol Oxidase (PPO, EC 1.10.3.1)

The polyphenol oxidase activity was estimated according to the method proposed by Kumar and Khan [26]. For the estimation of the enzyme, 1 mL of phosphate buffer, 0.5 mL of catechol, and 0.25 mL of enzyme extract were added to the cuvette and it was incubated for 2 min. The reaction was initiated by the addition of 0.5 mL of H_2_SO_4_ and any change in absorbance was noted at 495 nm for 1 min. One unit of enzyme activity is defined as the quantity of enzyme necessary to oxidize 1 µM of catechol. The activities of the enzyme were expressed as UA mg g^−1^ protein.

#### 2.7.9. Glutathione-S-Transferase (GST, EC 2.5.1.13)

The glutathione-S-transferase enzyme activity was estimated according to the method given by Habig and Jakoby [27]. The 3-mL reaction mixture in the cuvette contained 0.2 M phosphate buffer, 1 mM each of GSH and 1-Chloro-2,4-dinitrobenzene (CDNB), and the reaction was set off by adding 70 μLof enzyme extract. The increase in absorbance at 340 nm was recorded for 1 min at 3-s intervals. One unit of enzyme activity is defined as the quantity of enzyme catalyzing the development of 1 µM of conjugated GSH-CDNB min^−1^ g^−1^ plant tissue at 25 °C. The activity was revealed as UA mg g^−1^ protein.

#### 2.7.10. Glutathione Peroxidase (GPOX, EC 1.11.1.7)

The glutathione peroxidase activity of fresh plant samples was analyzed according to the method given by Flohé and Günzler [28]. The composition of the 3-mL reaction mixture in the cuvette consisted of 50 mM phosphate buffer, 0.5 mM EDTA, 1 mM of GSH, 1 mM of sodium azide, 0.15 mM NADPH and 0.15 mM H_2_O_2_, and 75 µL of enzyme extract. A decrease in the absorbance due to oxidation of NADPH was measured for 1 min at 340 nm at 3-s intervals. One unit of enzyme activity is defined as the amount of enzyme required to oxidize 1.0 µM of NADPH min^−1^g^−1^ fresh tissue at 37 °C and the activities were revealed as UA mg g^−1^ protein.

### 2.8. Non-Enzymatic Antioxidants

#### 2.8.1. Ascorbic Acid Content

The ascorbic acid content was estimated by the method of Roe and Kuether [29]. First, 1 g of fresh plant sample was crushed in 3 mL of 50 mM Tris buffer (pH 10) using a pestle and mortar under chilled conditions. The homogenate was centrifuged at 13,000 rpm and 4 °C for 20 min. Then, 500 μL of supernatant were mixed with 4 mLof distilled water, 0.5 mL 50% TCA, and 100 mg of activated charcoal in test tubes. The mixture was thoroughly mixed and filtered through Whatman filter paper No. 1. To the filtrate, 0.4 mL of 2,4-Dinitrophenylhydrazine (DNPH) reagent was added and incubation was given at 37 °C for 3 h. Cooling was done using an ice bath followed by the addition of 1.6 mL of cold H_2_SO_4_ (65%). Again, incubation was given for 30 min at room temperature and the absorbance was taken at 520 nm. Ascorbic acid in the concentration of 1 mg/100 mL was used as a standard and its content in the sample was expressed as mg ascorbic acid g^−1^ fresh weight.

#### 2.8.2. Tocopherol Content

Tocopherol (vitamin E) was estimated by the method given by Martinek [30]. First, 1 g of fresh plant sample was crushed in 3 mL of 50 mM Tris buffer (pH 10) using a pestle and mortar under chilled conditions. The homogenate was centrifuged at 13,000 rpm and 4 °C for 20 min. Then, 500 μL of supernatant were mixed with 500 μL distilled water and 500 μL FeCl_3_ (0.12% in absolute ethanol) in test tubes followed by vigorous shaking until precipitates formed. The reaction mixture was mixed with 500 μL xylene and vortexed for 30 s. The solution was centrifuged for 10 min at 3000 rpm. The upper xylene layer was mixed with an equal volume of 2,4,6-Tripyridyl-S-triazine (TPTZ) (12% in n-propanol) and the absorbance was read at 600 nm. A 1 mg 100 mL^−1^ concentration of tocopherol was used as the standard tocopherol content in sample and was expressed as mg tocopherol g^−1 ^fresh weight.

#### 2.8.3. Glutathione Content

The GSH content was estimated by the method given by Sedlak and Lindsay [31]. First, 1 g of fresh plant sample was crushed in 3 mL of 0.2 M Tris buffer (pH 8.2) using a pestle and mortar under chilled conditions. The homogenate was centrifuged at 13,000 rpm and 4 °C for 20 min. Then, 100 μL of supernatant was mixed with 1 mL of 0.2 M Tris buffer (pH 8.2), 50 μL DTNB (5,5’-dithiobis- 2-nitrobenzoic acid), and 4 mL of absolute methanol in test tubes followed by an incubation of 15 min at room temperature. The reaction mixture was centrifuged for 15 min at 3000 rpm and the absorbance of the supernatant was noted at 412 nm. GSH at a concentration of 1mg 100^−1^ mL was used as a standard for determining the GSH content in the sample. The value was expressed as mg GSH g^−1^ fresh weight.

#### 2.8.4. Glutathione Imaging

Tagging of glutathione in the roots of *Vigna radiata *was done with MCB (monochlorobimane) dye according to the method of Hartmann et al. [32]. MCB produces a fluorescent adduct with GSH. In the meanwhile, sodium azide depletes adenosine triphosphate (ATP) from the cells in order to avoid vacuolar seizure of the MCB-GSH adduct caused by ATP. The root samples were dipped in the 25 μM MCB dye containing 5 mM sodium azide for about 15 to 20 min. Samples were then mounted in distilled water on a glass slide and covered with a cover slip. The slides were then observed under a confocal microscope at an excitation wavelength of 351–364 nm and emission wavelength of 477 nm. The intensity of the blue color indicates the measure of glutathione in the roots.

### 2.9. Polyphenol Estimation

Qualitative as well as quantitative analysis of the plant samples for polyphenolic compounds was carried out using ultra-high-performance liquid chromatography (UHPLC). First, 500 mg of fresh plant material were crushed in 2 mL of high-performance liquid chromatography (HPLC)-grade methanol using a pestle and mortar. The solution was centrifuged at 13,000 rpm for 20 min and filtered using 0.2-micron filter paper. The portrayal of phenolic compounds was executed using a 130 MPa Shimadzu UHPLC (Nexera) system, equipped with a DGU-20As prominence degasser, LC-30AD liquid chromatograph, SIL-30AC autosampler, CTO-10AS VP column oven, CBM-20A communications bus module, and SPD-M20A photodiode array detector (PDA). The chromatography was carried out at room temperature with a flow rate of 1 mL/min at λ 280 nm. A 150 × 4.6 mm C-18 column with a pore size of 5 µm was used. The injection volume was 5 µL. The solvent system comprised of solvent A (0.01% acetic acid in water) and solvent B (methanol). The gradient runs were 70% A and 30% B, reaching 45% B at 12 min, 75% B at 13.5 min, 75% B at 15 min, 50% B at 16.6 min, 25% B at 18 min, 25% B at 20 min, 30% B at 21 min, and stopped at 22 min, with an elution of 4 min. The mixture of 11 polyphenolic standards, namely gallic acid (C_7_H_6_O_5_), catechin (C_15_H_14_O_6_), chlorogenic acid (C_16_H_18_O_9_), epicatechin (C_15_H_14_O_6_), caffeic acid (C_9_H_8_O_4_), umbelliferone (C_9_H_6_O_3_), coumaric acid (C_9_H_8_O_3_), rutin (C_27_H_30_O_16_), ellagic acid (C_14_H_6_O_8_), quercetin (C_15_H_10_O_7_), and kaempferol (C_15_H_10_O_6_), was diluted with methanol at different concentrations by serial dilution for quantitative analysis. The calibration curves were generated by plotting the concentrations versus peak areas. The detection of every compound was based on a combination of the retention time and spectral similarity. The detection and quantification limit for all the detected compounds were calculated on the basis of signal-to-noise ratio (S/N) of 3 and 10 with the corresponding standard solution, respectively.

### 2.10. Amino Acid Profiling

The amino acid analysis was done using an amino acid analyzer by making slight modifications to the method given by Iriti et al. [33]. First, 0.25 mg of fresh plant sample were crushed in 1.25 mL of 80% methanol using a pestle and mortar in chilled conditions. The homogenized sample was centrifuged for 20 min at 13,000 rpm. The supernatant was mixed with 6% sulfosalicylic acid in a ratio of 1:1 and the solution was further diluted with 0.1 N HCl in a ratio of 1:4. The solution was filtered using 0.2-micron filter paper. Using an auto sampler, 1 µL of sample was injected into the C-18 silica-bonded, 150-mm-long amino acid column with a pore size of 120 Å and particle size of 5 µm. The amino acid analysis was carried out using a Shimadzu, Nexera X_2_ amino acid analyzer. A 150-mm-long C-18 silica-bonded column with a 120 Å pore size and 5 micrometer particle size was used for the analysis of amino acids. The injection volume was 1 µL, run time was 32 min, oven temperature was 40 °C, and pump flow rate was 1 mL min^−1^. Mobile phase “A” consisted of 5.6 pH phosphate buffer; mobile phase “B” comprised of ultra-pure water, methanol, and acetonitrile in the ratio of 15:40:45. The detection of every amino acid was based on a combination of the retention time and spectral similarity. The detection and quantification limits for all the detected amino acids were calculated on the basis of the signal-to-noise ratio (S/N) of 3 and 10 with the corresponding standard solution, respectively.

### 2.11. Organic Acid Profiling

Organic acids in the test samples of 7-day-old *Vigna radiata* plants were estimated using GC-MS according to the method given by Chen et al. [34]. The 7-day-old *Vigna radiata* plants were washed with distilled water, dried at room temperature, and crushed to make powder with the help of a pestle and mortar. Then, 50 mg of powder of the dried plant sample was mixed with 500 µL of 0.5N HCl and 500 µL of methanol. The mixture was shaken for 3 h followed by centrifugation for 10 min at 10,000× *g. *To the supernatant, 300 µL of methanol and 100 µL of 50% H_2_SO_4_ were added and overnight incubation was given at 60 °C using a water bath. The solution was cooled to 25 °C followed by the addition of 800 µL of chloroform and 400 µL of distilled water. The solution was subjected to vortexing for one minute and the lower chloroform layer was used for the assessment of organic acids. To estimate the content of organic acids, 2 µL of the chloroform layer were injected into the GC-MS system (Shimadzu GC-MS-QP2010 Plus) in split mode. A DB-5 ms analytical column was used and the initial column temperature for one minute was 50 °C, which was raised to 125 °C at a rate of 25 °C min^−1^ followed by a further increase to 300 °C at a rate of 10 °C/min and held for 15 min. The injection temperature was 250 °C, the carrier gas used was helium, and the gas flow in the column was 1.7 mL min^−1^. The ion source temperature and interface temperature was set at 200 °C and 280 °C, respectively. The quantification of organic acids was done using a standard curve.

### 2.12. Statistical Analysis

All experimental measurements were carried out in triplicate. The mean values and standard error were calculated. The data were analyzed statistically by two-way analysis of variance (ANOVA), as described by Bailey [35]. Tukey’s multiple comparison test was used to compare the difference among means by using the honest significant difference HSD values. Comparisons with *P*-values ≤ 0.05 were considered as significantly different. Multiple regression analysis with interaction (MLR) was carried out to identify the nature of the effect brought about by independent variables Cr, MEL, and their interaction (Cr(VI) × MEL). β-regression values provided the relative effects of the independent variables:

X_1_ = Cr(VI), X_2_ = MEL and X_1_X_2_ = Cr(VI) × MEL.

## 3. Results

### 3.1. Phenolic Pigments

The data obtained on alterations in the contents of anthocyanin and xanthophylls pigments due to the effect of different treatments of Cr(VI), MEL, and their interaction in *Vigna radiata* plants are presented in Table 2. It was noted that in relation to 1.2 µg anthocyanin g^−1^ FW in control plants, the anthocyanin content in 7-day-old plants of *Vigna radiata* increased to 13.75 µg g^−1^ FW with the Cr(VI) metal treatment. MEL application to Cr(VI)-treated plants further enhanced the content and the plants grown in combination with Cr(VI) and 500 ppm MEL showed a maximum increase of 36.61 µg g^−1^ FW in the anthocyanin content. Various treatments had a significant role in modulating the increase of the xanthophylls pigment. The xanthophyll content in 7-day-old Cr(VI) metal-stressed plants was elevated to 28.81 µg g^−1^ FW in contrast to 16.1 µg g^−1^ FW in control plants. The exogenous supplementation of MEL at the 125, 250, and 500 ppm concentrations to Cr(VI)-stressed plants further enhanced the xanthophyll content to 34.95, 42.37, and 48.94 µg g^−1^ FW, respectively. Anthocyanin (HSD 5.92) and xanthophylls (HSD 5.67) showed a statistically significant difference in the two-way ANOVA analysis. Positive β values in the MLR analysis indicated the positive effect of Cr(VI), and the interaction of Cr(VI) × MEL on the anthocyanin and xanthophyll contents.

### 3.2. Protein Content

Table 3 represents the variations in the protein content of 7-day-old *Vigna radiata* plants due to Cr(VI) treatment and MEL supplementation. The protein content in untreated plants was observed to be 18.70 mg g^−1^ FW, which lowered to 9.60 mg g^−1^ FW in the Cr(VI)-treated plants. The supplementation of 125, 250, and 500 ppm MEL concentrations to the Cr(VI)-treated plants resulted in the elevation of the protein content to 12.60, 15.73, and 16.83 mg g^−1^ FW, respectively. The ANOVA analysis depicted a statistically significant difference in the protein content, with an HSD value of 1.57 (Table 4). The MLR analysis revealed the negative impact of Cr(VI) treatment (negative β value) and positive impact of the Cr(VI) × MEL application (positive β value) on the protein content.

### 3.3. Enzymatic Antioxidants

The antioxidant enzymes were significantly altered by Cr(VI) exposure and supplementation with various concentrations of MEL. The superoxide dismutase (SOD) activity of 7-day-old *Vigna radiata *plants was enhanced from 1.37 (control) to 6.08 UA mg g^−1^ protein under Cr(VI) treatment (Table 3). The combination of Cr(VI) and MEL further increased the SOD activity, and maximum enhancement to 9.22 UA mg g^−1^ protein was noted in the case of Cr(VI) × 500 ppm MEL. Similar trends were observed in POD (Table 3), CAT, APOX, GR (Table 4), DHAR, PPO, GST, and GPOX (Table 5) as Cr(VI) increased the enzyme activity, and co-application of MEL further enhanced the enzyme activity.

Two-way ANOVA analysis revealed that all the enzymes, SOD, POD, CAT, APOX, GR, DHAR, PPO, GST, and GPOX, had a statistically significant difference except in the case of the F ratio of the Cr(VI) × MEL interaction in CAT activity. The HSD values for SOD, POD, CAT, APOX, GR, DHAR, PPO, GST, and GPOX were noted to be 1.51, 38.97, 5.24, 11.33, 8.63, 7.59, 1.29, 6.79, and 1.63, respectively. The β values in the MLR analysis for all the enzymes were positive for all the treatments, indicating the positive correlation.

### 3.4. Non-Enzymatic Antioxidants

The effect of individual and binary treatments of Cr(VI) and MEL on the contents of non-enzymatic antioxidants in 7-day-old *Vigna radiata* plants is presented in Table 6. The ascorbic acid content was observed to be enhanced to 22.6 µg g^−1^ FW in Cr(VI)-treated plants in response to 11.97 µg g^−1^ FW in the control. Supplementation with 500 ppm MEL to metal-stressed plants further elevated the ascorbic acid content to 49.05 µg g^−1^ FW. The tocopherol content in the plants was increased with Cr(VI) (47.88 µg g^−1^ FW) as compared to the control (21.88 µg g^−1^ FW). The content was further enhanced by the application of a combination of Cr(VI) and 125, 250 and 500 ppm MEL (62.52, 82.07, and 98.99 µg g^−1^ FW). The glutathione content was also observed to increase with Cr(VI) exposure to the plants (481.71 µg g^−1^ FW) as compared to the control (299.79 µg g^−1^ FW). Priming of metal-stressed plants with MEL elevated the glutathione content even higher (635.45 µg g^−1 ^FW at Cr(VI) × 500 ppm MEL). Ascorbic acid, tocopherol, and glutathione contents in 7-day-old *Vigna radiata* plants showed a statistically significant difference at *p *≤ 0.05 in the two-way ANOVA analysis, with HSD values of 3.42, 10.001, and 50.8, respectively. MLR analysis revealed that ascorbic acid, tocopherol, and glutathione increased (positive β values) with Cr(VI) treatment and Cr(VI) × MEL interactions.

### 3.5. Glutathione Imaging

**Figure 1:**
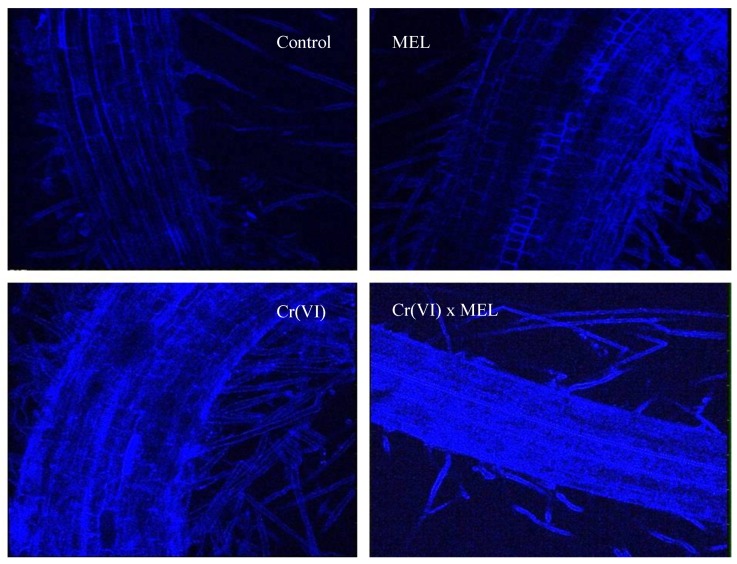
Effect of seed soaking treatment of methanol extract of *Rhododendron arboreum* leaves (MEL) on glutathione accumulation in *Vigna radiata* seedlings under Cr(VI) (250 µM) stress.

Tagging of glutathione in the roots of *Vigna radiata *was done by incubating the root sections in monochlorobimane dye (MCB) according to the method of Hartmann et al. [32] and the imaging was visualized using confocal microscopy. MCB produces a fluorescent adduct with glutathione. The intensity of the blue color indicates the measure of glutathione in roots. The intensity of the blue color indicates that the glutathione content increased with Cr(VI) stress as compared to the control plants. The amplification in the amount of blue color proves that co-application of MEL (500 ppm) along with Cr(VI) stress further increased the production of glutathione (Figure 1).

### 3.6. Polyphenol Content

Qualitative as well as quantitative analysis of the plant samples for polyphenolic compounds was carried out using ultra high-performance liquid chromatography (UHPLC). Five polyphenols, namely gallic acid, chlorogenic acid, caffeic acid, catechin, and coumaric acid, were found to be present in the plants. A comparative change in the polyphenolic contents of *Vigna radiata* plants grown under Cr(VI) exposure and the combination of Cr(VI) × MEL treatments is represented in Table 7. The contents of all the polyphenols increased with Cr(VI) stress. The supplementation of MEL to Cr(VI)-exposed plants further enhanced the polyphenol content and the maximum increase was observed in the case of the combination of Cr(VI) × 500 ppm MEL. Two-way ANOVA analysis of the polyphenols revealed a statistically significant difference. However, in case of catechin, the interaction of Cr(VI) × MEL treatment was noted to be non-significant. The β values from MLR analysis depict that gallic acid was positively affected by Cr(VI) treatment and negatively affected by co-application of Cr(VI) × MEL, whereas chlorogenic acid, caffeic acid, catechin, and coumaric acid were positively affected by the Cr(VI) and Cr(VI) × MEL treatments.

### 3.7. Amino Acid Content

The total amino acids content decreased to 591.47 µg g^−1 ^FW with Cr(VI) stress from 2504.69 µg g^−1^ FW in control plants. MEL recovered the amino acid content in the Cr(VI)-stressed plants, and the maximum recovery was observed to be 2208.89 µg g^−1^ FW in plants given the combination of 500 ppm MEL with Cr(VI). In total, 17 amino acids, namely aspartic acid, glutamine, β-alanine, lysine, glutamic acid, asparagines, serine, isoleucine, glycine, threonine, citrulline, arginine, GABA (gamma-aminobutyric acid ), cystine, ornithine, proline, and methionine, were found to be present in the plant samples. The variations in the contents of these amino acids due to treatment with Cr(VI) and MEL alone or in combination with each other are presented in Table 8, Table 9, Table 10 and Table 11.

Two-way analysis of the variance of the amino acids revealed that glutamine, β-alanine and lysine, asparagine, serine, isoleucine, threonine, arginine, cystine, and the total amino acids were statistically significant for the Cr(VI), MEL, and Cr(VI) × MEL treatments, whereas the F ratios of aspartic acid, glutamic acid, glycine, citrulline, GABA, ornithine, proline, and methionine were statistically significant for MEL and Cr(VI) × MEL but not for theCr(VI) treatment. MLR analysis of aspartic acid resulted in positive values of the β-regression coefficient for Cr(VI) treatment and positive values of the β-regression coefficients for the combined Cr(VI) × MEL treatment, which reveals that Cr(VI) stress decreased, whereas, Cr(VI) × MEL increased the aspartic acid content in the *Vigna radiata* plants. The glutamine, β-alanine, lysine (Table 8), glutamic acid, asparagine, serine, isoleucine (Table 9), glycine, threonine, citrulline, arginine, GABA (Table 10), cystine, ornithine, proline, methionine, and total amino acids (Table 11) revealed negative values of the β-regression coefficients for Cr(VI) treatment, indicating its negative effect, and positive values of the β-regression coefficients for the interaction of Cr(VI) × MEL, indicating the increasing effect in the amino acids.

### 3.8. Organic Acids

Data obtained on the effect of Cr(VI) exposure and the combination of MEL with Cr(VI) treatment on the organic acids of the Krebs cycle (citrate, succinate, fumarate, and malate) in *Vigna radiata* plants are presented in Table 12. The fumaric acid content was elevated to 0.415 mg g^−1^ DW (dry weight) in Cr(VI)-treated plants in comparison to control plants (0.38 mg g^−1^ DW). The 500 ppm MEL application to the Cr(VI)-treated plants enhanced the fumaric acid content to 0.416 mg g^−1^ DW. The citric acid content also increased to 2.94 mg g^−1^ DW in Cr(VI)-exposed plants as compared to 2.32 mg g^−1^ DW in unexposed plants. Treatment with 500 ppm MEL further enhanced its content to 3.68 mg g^−1^ DW in Cr(VI)-treated plants as compared to non-treated plants. An enhancement was observed in the malic acid content of Cr(VI)-treated plants to 2.15 mg g^−1^ DW in comparison with control plants, which contained 1.47 mg malic acid content g^−1^ DW. Co-application of different MEL concentrations (125, 250, and 500 ppm) to Cr(VI)-stressed plants resulted in an increase of the malic acid content to 2.19, 2.48, and 2.50 mg g^−1^ DW, respectively. The succinic acid content followed the same trend. The succinic acid content in control plants was observed to be 0.870 mg g^−1^ DW, which increased to 0.876 mg g^−1^ DW with Cr(VI) application. Supplementation with 125, 250, and 500 ppm MEL to Cr(VI)-exposed plants increased the succinic acid content to 0.867, 0.884, and 0.899 mg g^−1^ DW, respectively.

Two-way ANOVA revealed that the F ratios of all the four organic acids were statistically significant under Cr(VI) treatment, whereas the F ratio of the MEL dose was only significant in case of the citric acid content. F ratios for the interaction of Cr(VI) × MEL were statistically significant for the fumaric acid, citric acid, and succinic acid but not significant for the malic acid content. HSD values for fumaric acid, malic acid, citric acid, and succinic acid contents were noted to be 0.02, 0.61, 0.4, and 0.09, respectively. MLR analysis revealed the positive impact of Cr(VI) treatment (positive β values) and the negative impact of the interaction of Cr(VI) × MEL (positive β values) on thefumaric acid and succinate contents, whereas Cr(VI) and Cr(VI) × MEL had a positive effect (positive β values) on the malic acid and citric acid contents.

## 4. Discussion

Heavy metal contamination in soil is one of the foremost problems accountable for a reduction in agricultural yield. The most crucial segments of plant life are seed germination and growth of seedlings and these are adversely affected by metal stress [36]. Plants exposed to metal stress endure plentiful physiological and biochemical alterations because metal exposure involves oxidative stress [36]. The embarkment of stress endurance takes place when a stress causative agent approaches the cell surface or penetrates into the cytoplasm and injures the cell. The successive events intended to reinstate cell function are regarded as the stress response. The stress response is activated by a signal from a suitable receptor instantaneously after the commencement of the prevalence of the stress factor. At this stage, cell organization and utility is hindered. Oxidative damage generated by stress in the plant tissue is controlled by a combined operation of both antioxidant enzymes as well as non-enzymatic antioxidant systems. Apart from those, pigments, polyphenols, organic acids, and amino acids also play a key role in the plant defense against abiotic stress.

In the present study, the effect of Cr stress and MEL application on anthocyanin and xanthophyll pigments was observed. The results revealed that Cr treatment significantly enhanced the anthocyanin and xanthophyll content in comparison to untreated plants. Our results coincide with the findings of Kohli et al. [37], who observed an enhancement of anthocyanins and xanthophylls along with Pb metal stress in mustard, as well as Posmyk et al. [38], who reported an improvement in the anthocyanin content in red cabbage seedlings under Cu stress. The supplementation of MEL at 125, 250, and 500 ppm concentrations along with Cr(VI) metal stress further enhanced the pigment contents. Anthocyanins are involved in plants responses to various abiotic stresses and are reported to enhance stress tolerance by scavenging ROS or playing a role in stress signals. Kovinich et al. [39] reported that in response to various abiotic stresses in *Arabidopsis*, anthocyanins had diverse localizations at the tissue and organ levels. Anthocyanins amend the ROS level and the sensitivity to ROS-generating stresses in sustaining photosynthetic capacity [40]. Xanthophylls are considered as key antioxidants that defend plants subjected to heavy metal stress [41]. Xanthophyll is also vital for photo-protection; hence, its accumulation may downregulate Photosystem II actions to diminish oxidative damage [42]. Though reports explaining the approach to recover the pigment contents under stress are exclusively meager, the existing studies demonstrate that exogenous application of growth regulators improve the drought tolerance with increased activities of SOD, CAT, APX, ABA, and total improved pigment contents in maize [43]. The protein content in 7-day-old *Vigna radiata* Cr(VI)-stressed plants was observed to decrease as compared to the untreated control plants, whereas all the enzymes (SOD, POD, CAT, APOX, GR, DHAR, PPO, GST, and GPOX) in 7-day-old *Vigna radiata* plants showed enhanced activities under Cr(VI) stress. These results are in accordance with Rai et al. [44], who reported a decrease in protein and increase in enzymes with Cr stress. MEL supplementation showed an improvement in the protein content in Cr(VI)-treated plants. The combination of Cr(VI) and MEL further increased enzymatic activity at all concentrations when compared to Cr(VI) alone. SOD catalyzes the dismutation of the superoxide anion to di-oxygen and H_2_O_2_. Non-specific peroxidases are responsible for scavenging H_2_O_2_. Several reductases, like DHAR and GR, are responsible for keeping ascorbate and glutathione in the reduced form. SOD and catalase hold metal ions on their active sites as a fundamental fraction to combat the toxic effects of metal-stimulated ROS. The increase in both the protein content as well as enzymatic antioxidants supports the protective role of MEL towards the *Vigna radiata* plants to overcome the oxidative stress caused by Cr(VI). The non-enzymatic antioxidants, ascorbic acid, tocopherol, and glutathione, increased with Cr(VI) in comparison to untreated plants. The combination of Cr(VI) and MEL further enhanced the ascorbic acid, tocopherol, and glutathione contents and the maximum respective increase was observed in the case of Cr(VI) × 500 ppm MEL. Ascorbic acid is a well-known antioxidant involved in various processes, for example, cell wall expansion and cell division [45]. It scavenges free radicals directly in the aqueous phases of cells and guards the membrane and other hydrophobic sections from oxidative injury by redeveloping the antioxidant form of vitamin E [46]. It is also renowned for promoting photosynthetic pigments and improving the tolerance of plants in opposition to diverse stresses by scavenging ROS [47,48]. Therefore, the enhancement of anthocyanin and xanthophyll pigments in the present study might be attributed to the increased ascorbic acid content due to MEL application. Tocopherol is a water-soluble antioxidant found in chloroplasts that enhances pigment synthesis and modifies the biosynthesis pathways of pigments under stress conditions. Under salinity stress, it has been reported to play a role in several physiological functions, including growth regulation and the differentiation of plants [49,50]. Further, tocopherol has also been reported to protect cells from hydrogen peroxide and other free radicals generated in salinity stress by direct scavenging of ROS as well as collaborative action with antioxidant enzymes and other antioxidants [51,52,53]. An increase in the tocopherol content in *Arabidopsis thaliana* in response to Cd and Cu stress was observed [54]. The reason for this enhancement was the increase in transcripts encoding enzymes of the tocopherol biosynthetic pathway in response to metal exposure. The vitamin E-deficient (vte1) mutant was observed to be more prone to metal-induced stress compared to the wild-type (WT) control. It was concluded that tocopherol plays a vital function in the tolerance of *Arabidopsis* to oxidative stress induced by heavy metals, such as Cu and Cd [54]. Glutathione plays a central role in the scavenging of ROS as well as in the chelation of heavy metals. GSH protects the plants from heavy metal-induced oxidative damage by chelation, detoxification, and compartmentalization of heavy metals. Additionally, it acts through its metabolizing enzymes, particularly glutathione peroxidase, glutathione-*S*-transferase, glutathione reductase, and dehydroascorbate reductase, for efficient protection against ROS [55].

Polyphenols are proven stress busters for their ROS scavenging potential. Five polyphenols, namely gallic acid, chlorogenic acid, caffeic acid, catechin, and coumaric acid, were found to be present in the plants and a considerable increase in the levels of all polyphenolic compounds with Cr was observed in the present study. Kohli et al. [37] also established the increase in polyphenol contents under metal stress. This could be due to the increased activity of a variety of enzymes, such as chalcone synthase and cinnamate 4-hydroxylase, which are responsible for polyphenol synthesis [56,57]. In the present study, the combination of Cr(VI) and MEL further enhanced the polyphenol contents. It has been previously reported that antioxidants produced within plants reduce the effects of stresses in plants [58,59]. Those polyphenols possess the affinity to bind with metal ions and thereby inhibit the production of several ROS and some of them sequester the heavy metals entering the cell [60].

Amino acids are the building blocks of proteins. Different amino acids, namely aspartic acid, glutamine, β-alanine, lysine, glutamic acid, asparagine, serine, isoleucine, glycine, threonine, citrulline, arginine, GABA, cystine, ornithine, proline, and methionine, decreased with Cr(VI) stress when compared to control plants. This trend correlates with the decreased amount of protein in the present study. MEL recovered the amino acid content in the Cr(VI)-stressed plants. As compared to control plants, the contents of four organic acids (fumarate, citrate, malate, and succinate) were increased with Cr(VI) stress. The present study is in agreement with the work of Kohli et al. [61] and Ma [62], who reported that organic acids increase in abiotic stress conditions. Co-application of MEL along with Cr(VI) further increased the organic acid contents as compared to only Cr(VI)-treated plants, indicating the stress protective role of organic acids. Amino acids and organic acids are metabolites that have been reported to impart tolerance against heavy metal stress [63,64,65]. The utility of some of them is still unknown due to the convolution in plant responses to these stresses. In case of heavy metal stress, two mechanisms of detoxification and tolerance take place. Detoxification is external whereas tolerance is an internal process. In detoxification, plant roots secrete organic acids, which bind with metal ions to alter their movement as well as bioavailability, leading to the prevention of heavy metal uptake by plants. Meanwhile, in internal heavy metal tolerance, organic acids might chelate the metal ions inside the cytosol, converting them to less toxic or totally nontoxic byproducts [66,67]. Different plants have been reported to create a variety of ligands for aluminum, cadmium, copper, nickel, cobalt, and zinc. Amino acids and organic acids, such as citrate and malate, are reported to be probable ligands for heavy metals and are established to be engaged in their tolerance and detoxification [66,67,68,69]. Oven et al. [70] observed that cobalt metal treatment to a Co-hyperaccumulator, namely *Crotalaria cobalticola,* and non-accumulators, *Raufolia serpentina *and *Silene cucubalus*, resulted in an enhancement of citrate, indicating its participation in the heavy metal ion complex formation. Malate is reported to chelate zinc in the cytosols of Zn hyperaccumulators [71]. Zhang et al. [72] observed that taking away the aluminum from the roots leads to a quick decrease in malate release to the non-aluminum point, which demonstrated a receptive aluminum and malate-secreting mechanism. Over 24 h of exposure to 50 μM aluminum, 10-fold elevated malate and three- to five-fold elevated succinate secretion was observed in aluminum-tolerant genotypes as compared to aluminum-sensitive seedlings [73].

Ellagic acid has been reported by Ascacio-Valdés et al. [74] to have the ability to protect plants against stresses because of its antioxidant activity. Moreover, Khan et al. [75] reported that ellagic acid is one of the best antioxidants to shield *Brassica napus *L. plants against salinity stress, and due to its antioxidant properties, ellagic acid can enhance the growth and yield of the crop. *Rhododendron* leaf extract (MEL) is enriched with ellagic acid and several other antioxidant polyphenolic compounds. As a natural source of ellagic acid, it is as beneficial as pure ellagic acid for improving stress tolerance by regulating different physiological processes under Cr metal stress. Consequently, *Rhododendron* leaf extract can be used as an economical source of polyphenols, especially ellagic acid, for safeguarding plants from toxic effects of Cr stress.

## 5. Conclusions

In the present study, Cr stress brought about physiological as well as metabolic alterations in *Vigna radiata* plants even at small concentrations. The *Rhododendron* leaf extract supplementation to Cr-stressed *Vigna radiata* plants helps in tolerating Cr toxicity by modulating the contents of pigments and activation of the enzymatic as well as non-enzymatic antioxidativedefense system. It also restored polyphenols, organic acids, and amino acids, which also provides extra protection to the plants from Cr stress. We thus conclude that exogenous application of *Rhododendron* leaf extract (rich in ellagic acid) reduced the effect of chromium metal stress in *Vigna radiata* plants. Further prospects of the work will be intended towards fortification of the idea of the stress ameliorative activity of *Rhododendron* extract by investigating the mechanism attributed to the increased defense of plants against heavy metal stress.

## Figures and Tables

**Table 1:** Different treatments given to *Vigna radiata* in the present investigation.

Sr. No.	Name of the Treatment	MEL (ppm)	Cr(VI) (μM)
1.	Control	0	0
2.	MEL-1	125	0
3.	MEL-2	250	0
4.	MEL-3	500	0
5.	Cr(VI)	0	250
6.	Cr(VI) + MEL-1	125	250
7.	Cr(VI) + MEL-2	250	250
8.	Cr(VI) + MEL-3	500	250

**Table 2:** Effect of Cr(VI), different concentrations of methanol extract of *Rhododendron arboreum* leaves (MEL), and their combinations on anthocyanin and xanthophyll content in 7-day-old *Vigna radiata *seedlings. The values are the means of three replicates ± S.D. (standard deviation). Tukeys test performed and significance checked at *p ≤ 0.05. the F ratio is the term in which ANOVA is represented; Cr(VI) = Dose, MEL treatments = Treatment and combination of Cr(VI) and MEL = Dose × treatment.

**Concentrations**		**Anthocyanin (µg g^−1^ FW)**		**Xanthophyll (µg g^−1^ FW)**	
**Cr(VI) (µM)**	**MEL (ppm)**				
0	0	1.20 ± 0.58		16.10 ± 0.97	
0	125	2.78 ± 1.11		18.85 ± 1.32	
0	250	5.39 ± 1.12		22.24 ± 0.63	
0	500	11.89 ± 1.37		25.21 ± 1.94	
250	0	13.75 ± 0.42		28.81 ± 0.97	
250	125	17.47 ± 3.23		34.95 ± 2.54	
250	250	24.34 ± 3.20		42.37 ± 3.19	
250	500	36.61 ± 3.07		48.94 ± 2.77	
F-ratio Treatment _(1,16)_		429.96*		492.25*	
F-ratio Dose _(3,16)_		74.46*		60.38*	
F-ratio Treatment × Dose _(3,16)_		9.86*		8.55*	
HSD		5.92		5.67	
**Parameter**	**MLR Equation**	**β-Regression Coefficient**			**Multiple Correlation**
		**Cr(VI)**	**(MEL)**	**(Cr(VI) × MEL)**	
Anthocyanin	Y = 0.53 + 0.04 Cr(VI) + 0.02 (MEL) + 0.0001 (Cr(VI) × MEL)	0.54	0.36	0.38	0.99*
Xanthophyll	Y = 16.61 + 0.05 Cr(VI) + 0.01 (MEL) + 9 × 10^–5^ Cr(VI) × MEL)	0.61	0.31	0.34	0.99*

**Table 3:** Effect of Cr(VI), different concentrations of methanol extract of *Rhododendron arboreum* leaves (MEL), and their combinations on the protein content and activities of superoxide dismutase (SOD) and guaiacol peroxidase (POD) enzymes in 7-day-old *Vigna radiata* seedlings. The values are the means of three replicates ± S.D. (standard deviation). Tukeys test performed and significance checked at *p ≤ 0.05. The F ratio is the term in which ANOVA is represented; Cr(VI) = Dose, MEL treatments = Treatment and combination of Cr(VI) and MEL = Dose × treatment.

**Concentrations**		**Protein Content ** **(µg/g FW)**	**SOD ** **(UA mg g^−1^ Protein)**	**POD ** **(UA mg g^−1^ Protein)**	
**Cr(VI) (µM)**	**MEL (ppm)**				
0	0	18.70 ± 0.45	1.37 ± 0.11	132.08 ± 2.59	
0	125	14.23 ± 0.45	1.70 ± 0.47	132.35 ± 2.09	
0	250	15.90 ± 0.30	1.98 ± 1.24	133.38 ± 4.68	
0	500	16.50 ± 0.90	2.09 ± 0.05	146.67 ± 1.27	
250	0	9.60 ± 0.30	6.08 ± 0.66	160.58 ± 8.76	
250	125	12.60 ± 0.90	7.71 ± 0.08	184.77 ± 9.37	
250	250	15.73 ± 0.45	8.51 ± 0.17	196.39 ± 1.86	
250	500	16.83 ± 0.25	9.22 ± 0.09	241.91 ± 36.27	
F-ratio Treatment _(1,16)_		135.06*	779.27*	113.04*	
F-ratio Dose _(3,16)_		43.05*	14.63*	13.10*	
F-ratio Treatment × Dose _(3,16)_		93.06*	5.56*	6.05*	
HSD		1.57	1.51	38.97	
**Parameter**	**MLR Equation**	**β-Regression Coefficient**			**Multiple Correlation**
		**Cr(VI)**	**(MEL)**	**(Cr(VI) × MEL)**	
Protein Content	Y= 16.79 + −0.02 Cr(VI) + −0.002 (MEL) + 7 × 10^−6^ (Cr(VI) × MEL)	−1.17	−0.14	1.05	0.86*
SOD	Y= 1.48 + 0.02 Cr(VI) + 0.001 (MEL) +2 × 10^−6^ (Cr(VI) × MEL)	0.80	0.08	0.24	0.99*
POD	Y= 129.53 + 0.12 Cr(VI) + 0.03 (MEL) + 0.0005 (Cr(VI) × MEL)	0.43	0.15	0.59	0.99*

**Table 4:** Effect of Cr(VI), different concentrations of methanol extract of *Rhododendron arboreum* leaves (MEL), and their combinations on the activities of catalase (CAT), ascorbate peroxidase (APOX), and glutathione reductase (GR) enzymes in 7-day-old *Vigna radiata* seedlings. The values are the means of three replicates ± SD. (standard deviation). Tukeys test performed and significance checked at *p ≤ 0.05. The F ratio is the term in which ANOVA is represented; Cr(VI) = Dose, MEL treatments = Treatment and combination of Cr(VI) and MEL = Dose × treatment.

**Concentrations**		**CAT ** **(UA mg g^−1 ^Protein)**	**APOX ** **(UA mg g^−1 ^Protein)**		**GR ** **(UA mg g^−1 ^Protein)**
**Cr(VI) (µM)**	**MEL (ppm)**				
0	0	7.27 ± 1.19	14.28 ± 1.78		27.01 ± 3.62
0	125	7.56 ± 0.65	14.76 ± 4.32		25.96 ± 3.10
0	250	7.82 ± 1.86	15.02 ± 3.47		28.20 ± 2.26
0	500	8.40 ± 1.50	16.61 ± 2.82		28.44 ± 1.78
250	0	16.40 ±1.92	41.66 ± 6.27		47.16 ± 3.71
250	125	17.54 ± 2.19	53.04 ± 2.61		57.16 ± 1.24
250	250	18.58 ± 2.65	61.30 ± 5.74		61.44 ± 3.56
250	500	23.22 ± 2.06	80.94 ± 2.72		74.78 ± 3.92
F-ratio Treatment _(1,16)_		217.87*	725.75*		689.60*
F-ratio Dose _(3,16)_		5.24*	28.78*		24.07*
F-ratio Treatment × Dose _(3,16)_		2.76	22.64*		18.55*
HSD		5.24	11.33		8.63
**Parameter**	**MLR Equation**	**β-Regression Coefficient**			**Multiple Correlation**
		**Cr(VI)**	**(MEL)**	**(Cr(VI) × MEL)**	
CAT	Y= 7.27 + 0.03 Cr(VI) + 0.002 (MEL) + 5 × 10^−6^ (Cr(VI) × MEL)	0.73	0.07	0.33	0.99*
APOX	Y= 14.15 + 0.11 Cr(VI) + 0.004 (MEL) + 0.0003 (Cr(VI) × MEL)	0.57	0.03	0.51	0.99*
GR	Y= 26.54 + 0.08 Cr(VI) + 0.003 (MEL) + 0.0002 (Cr(VI) × MEL)	0.61	0.04	0.47	0.99*

**Table 5:** Effect of Cr(VI), different concentrations of methanol extract of *Rhododendron arboreum* leaves (MEL), and their combinations on the activities of dehydroascorbate reductase (DHAR), polyphenol oxydase (PPO), glutathione S-transferase (GST), and glutathione peroxidase (GPOX) enzymes in 7-day-old *Vigna radiata* seedlings. The values are the means of three replicates ± SD. (standard deviation). Tukeys test performed and significance checked at *p ≤ 0.05. The F ratio is the term in which ANOVA is represented; Cr(VI) = Dose, MEL treatments = Treatment and combination of Cr(VI) and MEL = Dose × treatment.

**Concentrations**		**DHAR ** **(UA mg g^−1 ^Protein)**	**PPO ** **(UA mg g^−1 ^Protein)**	**GST ** **(UA mg g^−1 ^Protein)**		**GPOX ** **(UA mg g^−1 ^Protein)**
**Cr(VI) (µM)**	**MEL (ppm)**					
0	0	26.95 ± 2.57	2.54 ± 0.42	16.80 ± 1.47		16.41 ± 0.34
0	125	25.71 ± 2.85	2.75 ± 0.27	16.91 ± 0.18		17.75 ± 0.39
0	250	27.80 ± 2.65	3.13 ± 0.13	17.90 ± 0.82		17.82 ± 0.13
0	500	30.57 ± 0.75	3.74 ± 0.37	18.56 ± 0.29		17.98 ± 0.34
250	0	54.28 ± 2.85	8.96 ± 0.86	34.64 ± 4.46		26.09 ± 0.67
250	125	69.62 ± 1.08	10.83 ± 0.43	45.82 ± 2.23		27.65 ± 0.79
250	250	78.92 ± 4.32	12.47 ± 0.43	56.23 ± 3.94		26.49 ± 0.21
250	500	92.65 ± 2.67	13.58 ± 0.37	64.51 ± 1.65		28.67 ± 1.05
F-ratio Treatment _(1,16)_		1770.58*	2035.90*	1115.51*		1708.56*
F-ratio Dose _(3,16)_		65.78*	45.55*	49.10*		13.93*
F-ratio Treatment × Dose _(3,16)_		44.24*	16.64*	38.32*		3.09*
HSD		7.59	1.29	6.79		1.63
**Parameter**	**MLR Equation**		**β-Regression Coefficient**			**Multiple Correlation**
			**Cr(VI)**	**(MEL)**	**(Cr(VI) x MEL)**	
DHAR	Y= 25.9 + 0.12 Cr(VI) + 0.008 (MEL) + 0.0003 (Cr(VI) × MEL)		0.63	0.06	0.44	0.99*
PPO	Y= 2.5 + 0.02Cr(VI) + 0.002 (MEL) +3 × 10^−6^ Cr(VI) × MEL)		0.79	0.10	0.25	0.99*
GST	Y= 16.71 + 0.08 Cr(VI) + 0.003 (MEL) + 0.0002 (Cr(VI) × MEL)		0.56	0.03	0.51	0.99*
GPOX	Y= 16.92 + 0.03 Cr(VI) + 0.002 (MEL) + 7 × 10^−7^ (Cr(VI) × MEL)		0.94	0.09	0.05	0.99*

**Table 6:** Effect of Cr(VI), different concentrations of methanol extract of *Rhododendron arboreum* leaves (MEL), and their combinations on ascorbic acid, tocopherol, and glutathione content in 7-day-old *Vigna radiata* seedlings. The values are the means of three replicates ± SD. (standard deviation). Tukeys test performed and significance checked at *p ≤ 0.05. The F ratio is the term in which ANOVA is represented; Cr(VI) = Dose, MEL treatments = Treatment and combination of Cr(VI) and MEL = Dose × treatment.

**Concentrations**		**Ascorbic Acid (µg g^−1^ FW)**	**Tocopherol (µg g^−1^ FW)**		**Glutathione (µg g^−1^ FW)**	
**Cr(VI) (µM)**	**MEL (ppm)**					
0	0	11.97 ± 0.36	21.88 ± 1.90		299.79 ± 7.68	
0	125	15.80 ± 0.72	30.42 ± 1.02		333.10 ± 8.87	
0	250	19.70 ± 1.88	32.22 ± 0.62		384.35 ± 20.33	
0	500	26.45 ± 0.49	40.76 ± 4.12		443.28 ± 16.01	
250	0	22.60 ± 0.41	47.88 ± 0.69		481.71 ± 11.74	
250	125	28.45 ± 1.27	62.52 ± 0.04		407.41 ± 20.33	
250	250	39.30 ± 0.65	82.07 ± 4.85		520.15 ± 29.10	
250	500	49.05 ± 2.23	98.99 ± 7.33		635.45 ± 19.34	
F-ratio Treatment _(1,16)_		1097.11*	828.52*		396.82*	
F-ratio Dose _(3,16)_		326.84*	107.15*		107.27*	
F-ratio Treatment × Dose _(3,16)_		32.78*	27.12*		13.43*	
HSD		3.42	10.001		50.8	
**Parameter**		**MLR Equation**	**β-Regression Coefficient**			**Multiple Correlation**
			**Cr(VI)**	**(MEL)**	**(Cr(VI) × MEL)**	
Ascorbic Acid		Y= 12.15 + 0.04 Cr(VI) + 0.02 (MEL) + 0.0001 (Cr(VI) × MEL)	0.47	0.46	0.37	0.99*
Tocopherol		Y= 23.59 + 0.1 Cr(VI) + 0.03 (MEL) + 0.0003 (Cr(VI) × MEL)	0.52	0.25	0.45	0.99*
Glutathione		Y= 301.33 + 0.51 Cr(VI) + 0.29 (MEL) + 0.0003 (Cr(VI) × MEL)	0.63	0.53	0.14	0.94*

**Table 7:** Effect of Cr(VI), different concentrations of methanol extract of *Rhododendron arboreum* leaves (MEL), and their combinations on polyphenol contents in 7-day-old *Vigna radiata* seedlings. The values are the means of three replicates ± S.D. (standard deviation). Tukeys test performed and significance checked at *p ≤ 0.05. The F ratio is the term in which ANOVA is represented; Cr(VI) = Dose, MEL treatments = Treatment and combination of Cr(VI) and MEL = Dose × treatment.

**Concentrations**		**Gallic Acid ** **(µg g^−1^ FW)**	**ChlorogenicAcid ** **(µg g^−1^ FW)**	**Caffeic Acid ** **(µg g^−1^ FW)**	**Catechin ** **(µg g^−1^ FW)**		**Coumaric Acid ** **(µg g^−1^ FW)**
**Cr(VI) (µM)**	**MEL (ppm)**						
0	0	9.60 ± 1.40	1.74 ± 0.05	0.10 ± 0.002	263.80 ± 16.15		0.18 ± 0.004
0	125	9.87 ± 0.60	1.64 ± 0.01	0.15 ± 0.008	268.03 ± 49.93		0.16 ± 0.006
0	250	10.46 ± 1.06	1.74 ± 0.03	0.16±0.003	282.89 ± 55.04		0.18 ± 0.006
0	500	13.57 ± 0.91	1.81 ± 0.03	0.18 ± 0.004	287.84 ± 9.55		0.21 ± 0.006
250	0	16.05 ± 0.43	4.60 ± 0.11	0.94 ± 0.009	331.08 ± 53.67		0.44 ± 0.020
250	125	13.22 ± 0.71	3.21 ± 0.64	0.84 ± 0.01	422.96 ± 10.10		0.35 ± 0.010
250	250	15.18 ± 0.61	4.22 ± 0.25	0.96 ± 0.04	430.51 ± 20.83		0.59 ± 0.020
250	500	17.51 ± 0.50	5.26 ± 0.12	1.24 ± 0.16	447.50 ± 35.40		0.74 ± 0.050
F-ratio Treatment _(1,16)_		185.09*	618.21*	124.54*	79.75*		1207.79*
F-ratio Dose _(3,16)_		24.66*	19.73*	16.50*	4.35*		87.41*
F-ratio Treatment × Dose _(3,16)_		3.93*	14.41*	10.08*	2.17		59.07*
HSD		2.35	0.72	0.16	102.71		0.06
**Parameter**		**MLR Equation**		**β-Regression Coefficient**			**Multiple Correlation**
				**Cr(VI)**	**(MEL)**	**(Cr(VI) × MEL)**	
Gallic Acid		Y= 9.08 + 0.02 Cr(VI) + 0.008 (MEL) + 1 × 10^−6^ (Cr(VI) × MEL)		0.96	0.54	−0.2	0.93*
Chlorogenic Acid		Y= 1.69 + 0.008 Cr(VI) + 0.0002 (MEL) + 8 × 10^−7^ (Cr(VI) × MEL)		0.76	0.02	0.24	0.94*
Caffeic Acid		Y= 0.12 + 0.002 Cr(VI) + 0.0001 (MEL) + 2× 10^−7^ (Cr(VI) × MEL)		0.83	0.06	0.21	0.99*
Catechin		Y= 264.5 + 0.39 Cr(VI) + 0.05 (MEL) + 0.0006 (Cr(VI) × MEL)		0.67	0.12	0.34	0.96*
Coumaric Acid		Y= 0.17 + 0.0008 Cr(VI) + 7 × 10^−6^ (MEL) + 3 × 10^−7^ (Cr(VI) × MEL)		0.51	0.05	0.53	0.96*

**Table 8:** Effect of Cr(VI), different concentrations of methanol extract of *Rhododendron arboreum* leaves (MEL), and their combinations on aspartic acid, glutamine, β-alanine, and lysine contents in 7-day-old *Vigna radiata* seedlings. The values are the means of three replicates ± SD. (standard deviation). Tukeys test performed and significance checked at *p ≤ 0.05. The F ratio is the term in which ANOVA is represented; Cr(VI) = Dose, MEL treatments = Treatment and combination of Cr(VI) and MEL = Dose × treatment.

**Concentrations**		**Aspartic Acid (µg g^−1^ FW)**	**Glutamine (µg g^−1^ FW)**	**β-Alanine (µg g^−1^ FW)**		**Lysine (µg g^−1^ FW)**
**Cr(VI) (µM)**	**MEL (ppm)**					
0	0	79.00 ± 0.85	40.61 ± 0.35	168.83 ± 0.86		35.11 ± 0.48
0	125	45.71 ± 2.43	37.30 ± 0.46	90.79 ± 0.34		17.81 ± 0.62
0	250	63.18 ± 0.39	38.45 ± 0.50	101.34 ± 0.51		18.85 ± 0.85
0	500	74.18 ± 0.68	39.72 ± 0.98	114.37 ± 0.48		20.73 ± 0.81
250	0	32.16 ± 0.60	8.82 ± 1.12	35.96 ± 0.98		18.54 ± 1.44
250	125	73.39 ± 0.57	30.08 ± 0.71	115.62 ± 0.22		23.70 ± 1.03
250	250	75.90 ± 0.13	36.40 ± 0.51	124.54 ± 1.40		29.54 ± 0.78
250	500	78.04 ± 0.51	39.60 ± 0.57	164.09 ± 0.67		30.84 ± 1.14
F-ratio Treatment _(1,16)_		2.43	1295.79*	769.44*		43.96*
F-ratio Dose _(3,16)_		509.11*	529.81*	2956.89*		48.24*
F-ratio Treatment × Dose _(3,16)_		1520.74*	655.19*	17456.18*		285.57*
HSD		2.87	1.98	2.19		2.64
**Parameter**		**MLR equation**	**β-Regression Coefficient**			**Multiple correlation**
			**Cr(VI)**	**(MEL)**	**(Cr(VI) × MEL)**	
Aspartic Acid		Y= 63.48 + −0.06 Cr(VI) + 0.009 (MEL) + 0.0003 (Cr(VI) × MEL)	−0.17	0.1	0.7	0.62
Glutamine		Y= 39.03 + −0.08 Cr(VI) + 5 × 10^−6^ (MEL) +0.0002 (Cr(VI) × MEL)	−1.12	−0.0009	0.94	0.88*
β-Alanine		Y= 135.01 + −0.3 Cr(VI) + −0.074 (MEL) + 0.0012 (Cr(VI) × MEL)	−0.95	−0.34	1.31	0.8*
Lysine		Y= 27.81 + −0.03 Cr(VI) + −0.02 (MEL) + 0.0002 (Cr(VI) × MEL)	−0.6	−0.64	1.26	0.71*

**Table 9:** Effect of Cr(VI), different concentrations of methanol extract of *Rhododendron arboreum* leaves (MEL), and their combinations on glutamic acid, asparagine, serine, and isoleucine contents in 7-day-old *Vigna radiata* seedlings. The values are the means of three replicates ± S.D. (standard deviation). Tukeys test performed and significance checked at *p ≤ 0.05. The F ratio is the term in which ANOVA is represented; Cr(VI) = Dose, MEL treatments = Treatment and combination of Cr(VI) and MEL = Dose × treatment.

**Concentrations**		**Glutamic Acid (µg g^−1^ FW)**	**Asparagine (µg g^−1^ FW)**	**Serine (µg g^−1^ FW)**		**Isoleucine (µg g^−1^ FW)**
**Cr(VI) ** **(µM)**	**MEL (ppm)**					
0	0	29.83 ± 0.76	1134.65 ± 48.39	10.01 ± 0.38		174.95 ± 1.75
0	125	15.84 ± 0.79	443.42 ± 9.80	1.23 ± 0.09		65.96 ± 0.77
0	250	18.80 ± 0.87	652.13 ± 14.26	1.94 ± 0.13		68.31 ± 0.60
0	500	22.84 ± 0.70	686.02 ± 12.23	3.68 ± 0.56		86.11 ± 1.10
250	0	8.65 ± 1.31	355.18 ± 34.14	0.58 ± 0.15		13.75 ± 3.03
250	125	25.35 ± 0.71	824.64 ± 26.16	6.15 ± 0.06		87.03 ± 1.25
250	250	27.43 ± 0.52	875.57 ± 16.85	6.98 ± 0.12		104.83 ± 0.99
250	500	28.47 ± 0.50	974.98 ± 22.29	7.84 ± 0.89		146.25 ± 16.09
F-ratio Treatment _(1,16)_		3.83	7.20*	49.47*		20.52*
F-ratio Dose _(3,16)_		73.33*	58.91*	30.23*		49.36*
F-ratio Treatment × Dose _(3,16)_		489.49*	651.33*	451.69*		447.64*
HSD		2.29	73.65	1.153		16.62
**Parameter**		**MLR equation**	**β-Regression Coefficient**			**Multiple correlation**
			**Cr(VI)**	**(MEL)**	**(Cr(VI) × MEL)**	
Glutamic Acid		Y= 23.43 + −0.03 Cr(VI) + −0.007 (MEL) + 0.0002 (Cr(VI) × MEL)	−0.61	−0.19	1.02	0.66
Asparagine		Y= 851.39 + −1.31 Cr(VI) + −0.55 (MEL) + 0.0065 (Cr(VI) × MEL)	−0.66	−0.42	1.13	0.64
Serine		Y= 6.15 + −0.01 Cr(VI) + −0.009 (MEL) + 9 × 10^−6^ (Cr(VI) × MEL)	−0.54	−0.5	1.13	0.64
Isoleucine		Y= 127.8 + −0.37 Cr(VI) + −0.13 (MEL) + 0.001 (Cr(VI) × MEL)	−0.99	−0.52	1.37	0.78*

**Table 10:** Effect of Cr(VI), different concentrations of methanol extract of *Rhododendron arboreum* leaves (MEL), and their combinations on glycine, threonine, citrulline, arginine, and gamma-aminobutyric acid (GABA) contents in 7-day-old *Vigna radiata* seedlings. The values are the means of three replicates ± SD. (standard deviation). Tukeys test performed and significance checked at *p ≤ 0.05. The F ratio is the term in which ANOVA is represented; Cr(VI) = Dose, MEL treatments = Treatment and combination of Cr(VI) and MEL = Dose × treatment.

**Concentrations**		**Glycine (µg g^−1^ FW)**	**Threonine (µg g^−1^ FW)**	**Citrulline (µg g^−1^ FW)**	**Arginine (µg g^−1^ FW)**		**GABA (µg g^−1^ FW)**
**Cr(VI)(µM)**	**MEL (ppm)**						
0	0	9.47 ± 0.56	6.39 ± 0.68	22.55 ± 1.18	647.02 ± 12.48		5.009 ± 0.26
0	125	4.64 ± 0.40	2.72 ± 0.22	10.97 ± 0.81	97.11 ± 5.87		1.89 ± 0.04
0	250	5.48 ± 0.46	3.38 ± 0.72	12.60 ± 0.50	164.57 ± 6.72		2.47 ± 0.40
0	500	6.07 ± 0.76	3.81 ± 0.06	15.61 ± 1.07	181.85 ± 6.55		3.06 ± 0.67
250	0	3.58 ± 0.32	1.37 ± 0.42	4.91 ± 0.75	78.43 ± 7.27		1.18 ± 0.37
250	125	6.77 ± 0.37	3.72 ± 0.09	15.93 ± 0.82	311.96 ± 49.42		2.89 ± 0.12
250	250	7.25 ± 0.29	4.22 ± 0.27	19.47 ± 0.62	466.28 ± 18.01		3.49 ± 0.43
250	500	8.14 ± 0.96	4.86 ± 0.76	20.94 ± 0.52	575.68 ± 11.60		4.16 ± 0.83
F-ratio Treatment _(1,16)_		0.006	7.06*	0.12	108.4*		0.84
F-ratio Dose _(3,16)_		6.24*	5.26*	45.23*	91.77*		6.97*
F-ratio Treatment × Dose _(3,16)_		73.37*	56.22*	305.2*	725.5*		41.19*
HSD		1.59	1.38	2.32	56.87		1.31
**Parameter**		**MLR Equation**		**β-Regression Coefficient**			**Multiple Correlation**
				**Cr(VI)**	**(MEL)**	**(Cr(VI) × MEL)**	
Glycine		Y= 7.42 + −0.01 Cr(VI) + −0.005 (MEL) + 5 × 10^−6^ (Cr(VI) × MEL)		−0.77	−0.47	1.2	0.67
Threonine		Y= 4.84 + −0.01 Cr(VI) + −0.003 (MEL) + 4 × 10^−6^ (Cr(VI) × MEL)		−0.96	−0.46	1.19	0.7
Citrulline		Y= 17.32 + −0.03 Cr(VI) + −0.009 (MEL) + 0.0001 (Cr(VI) × MEL)		−0.76	−0.29	1.17	0.72*
Arginine		Y= 423.61 + −1.09 Cr(VI) + −0.69 (MEL) + 0.006 (Cr(VI) × MEL)		−0.65	−0.61	1.34	0.76*
GABA		Y= 3.64 + −0.008 Cr(VI) + −0.002 (MEL) + 3 × 10^−6^ (Cr(VI) × MEL)		−0.83	−0.39	1.18	0.69

**Table 11:** Effect of Cr(VI), different concentrations of methanol extract of *Rhododendron arboreum* leaves (MEL), and their combinations on cystine, ornithine, proline, methionine, and total amino acid contents in 7-day-old *Vigna radiata* seedlings. The values are the means of three replicates ± SD. (standard deviation). Tukeys test performed and significance checked at *p ≤ 0.05. The F ratio is the term in which ANOVA is represented; Cr(VI) = Dose, MEL treatments = Treatment and combination of Cr(VI) and MEL = Dose × treatment.

**Concentrations**		**Cystine (µg g^−1^ FW)**	**Ornithine (µg g^−1^ FW)**	**Proline (µg g^−1^ FW)**	**Methionine (µg g^−1^ FW)**		**Total Amino Acids (µg g^−1^ FW)**
**Cr(VI) (µM)**	**MEL (ppm)**						
0	0	28.42 ± 2.48	9.91 ± 1.03	44.49 ± 9.005	51.98 ± 0.79		2504.69 ± 54.9
0	125	8.56 ± 2.02	5.82 ± 0.92	14.39 ± 2.14	26.21 ± 0.55		893.15 ± 24.40
0	250	11.94 ± 1.48	6.58 ± 0.44	16.94 ± 0.90	33.15 ± 1.47		1223.58 ± 13.29
0	500	14.78 ± 0.85	7.38 ± 0.51	19.38 ± 0.59	43.61 ± 0.67		1347.07 ± 14.95
250	0	4.54 ± 0.54	3.30 ± 0.47	9.99 ± 0.92	9.10 ± 0.68		591.47 ± 47.18
250	125	19.49 ± 0.64	7.47 ± 0.40	22.49 ± 1.65	45.85 ± 1.04		1626.33 ± 73.72
250	250	23.03 ± 0.43	8.42 ± 0.19	26.66 ± 2.99	47.92 ± 1.009		1892.25 ± 29.66
250	500	27.77 ± 1.03	9.59 ± 1.17	32.17 ± 3.34	50.54 ± 0.52		2208.89 ± 49.85
F-ratio Treatment _(1,16)_		24.46*	0.59	0.41	1.08		24.5*
F-ratio Dose _(3,16)_		28.71*	9.01*	6.87*	366.005*		144.16*
F-ratio Treatment × Dose _(3,16)_		250.58*	52.11*	54.57*	1549.31*		1424.96*
HSD		3.89	2.04	10.52	2.53		122.65
**Parameter**		**MLR Equation**		**β-Regression Coefficient**			**Multiple correlation**
				**Cr(VI)**	**(MEL)**	**(Cr(VI) × MEL)**	
Cystine		Y= 19.91 + −0.04 Cr(VI) + −0.01 (MEL) + 0.0002 (Cr(VI) × MEL)		−0.62	−0.4	1.23	0.74*
Ornithine		Y= 8.11 + −0.01 Cr(VI) + −0.003 (MEL) + 6 × 10^−6^ (Cr(VI) × MEL)		−0.84	−0.29	1.22	0.76*
Proline		Y= 31.96 + −0.07 Cr(VI) + −0.03 (MEL) + 0.0003 (Cr(VI) × MEL)		−0.87	−0.66	1.29	0.7*
Methionine		Y= 39.58 + −0.06 Cr(VI) + −0.004 (MEL) + 0.0003 (Cr(VI) × MEL)		−0.58	−0.05	0.89	0.65
Total Amino Acids		Y= 1835.4 + −3.57 Cr(VI) + −1.56 (MEL) + 0.01 (Cr(VI) × MEL)		−0.73	−0.47	1.25	0.71*

**Table 12:** Effect of Cr(VI), different concentrations of methanol extract of *Rhododendron arboreum* leaves (MEL), and their combinations on fumaric acid, malic acid, citric acid, and succinic acid content in 7-day-old *Vigna radiata* seedlings. The values are the means of three replicates ± SD. (standard deviation). Tukeys test performed and significance checked at *p ≤ 0.05. The F ratio is the term in which ANOVA is represented; Cr(VI) = Dose, MEL treatments = Treatment and combination of Cr(VI) and MEL = Dose × treatment.

**Concentrations**		**FumaricAcid Content ** **(µg g^−1^ DW)**	**Malic Acid Content ** **(µg g^−1^ DW)**	**Citric Acid Content ** **(µg g^−1^ DW)**		**Succinic Acid Content ** **(µg g^−1^ DW)**
**Cr(VI)(µM)**	**MEL (ppm)**					
0	0	0.38 ± 0.009	1.47 ± 0.15	2.32 ± 0.02		0.870 ± 0.05
0	125	0.393 ± 0.005	1.45 ± 0.41	2.32 ± 0.15		0.783 ± 0.01
0	250	0.395 ± 0.005	1.46 ± 0.19	2.40 ± 0.01		0.824 ± 0.01
0	500	0.410 ± 0.008	1.47 ± 0.11	2.40 ± 0.01		0.866 ± 0.08
250	0	0.415 ± 0.010	2.15 ± 0.21	2.94 ± 0.34		0.876 ± 0.03
250	125	0.394 ± 0.008	2.19 ± 0.23	3.64 ± 0.10		0.867 ± 0.04
250	250	0.409 ± 0.007	2.48 ± 0.02	3.67 ± 0.05		0.884 ± 0.04
250	500	0.416 ± 0.008	2.50 ± 0.15	3.68 ± 0.08		0.899 ± 0.01
F-ratio Treatment _(1,16)_		10.75*	95.55*	370.78*		11.30*
F-ratio Dose _(3,16)_		1.97	1.11	11.18*		1.07
F-ratio Treatment × Dose _(3,16)_		10.73*	1.05	8.20*		3.71*
HSD		0.02	0.61	0.4		0.09
**Parameter**		**MLR Equation**	**β-Regression Coefficient**			**Multiple Correlation**
			**Cr(VI)**	**(MEL)**	**(Cr(VI) × MEL)**	
Fumaric acid		Y= 0.38 + 0.0001 Cr(VI) + 5 × 10^−6^ (MEL) + 3 × 10^−8^ (Cr(VI) × MEL)	1.21	0.78	−1.16	0.8*
Malic acid		Y= 1.46 + 0.002 Cr(VI) + 1 × 10^−6^ 0.000001 (MEL) + 3 × 10^−7^ (Cr(VI) × MEL)	0.78	0.006	0.28	0.99*
Citric acid		Y= 2.23 + 0.003 Cr(VI) + 0.0002 (MEL) + 4 × 10^−7^ (Cr(VI) × MEL)	0.74	0.05	0.28	0.96*
Succinic acid		Y= 0.82 + 0.0002 Cr(VI) + 4 × 10^−6^ 0.000004 (MEL) + 3 × 10^−8^ (Cr(VI) × MEL)	0.88	0.21	−0.34	0.68
